# Loop dynamics, allostery, and function in protein tyrosine phosphatases: insights from molecular simulations

**DOI:** 10.1042/BST20250018

**Published:** 2026-01-21

**Authors:** Colin L. Welsh, Shina Caroline Lynn Kamerlin

**Affiliations:** 1 School of Chemistry and Biochemistry, Georgia Institute of Technology, 901 Atlantic Drive NW, Atlanta, Georgia, 30332-0400, U.S.A; 2 School of Chemical and Biomolecular Engineering, Georgia Institute of Technology, 311 Ferst Drive, Atlanta, GA, 30332, U.S.A.; 3 Department of Chemistry, Lund University, Box 124, Lund, 22100, Sweden

**Keywords:** biomolecular simulations, enzyme loop dynamics, protein allostery, protein conformational changes, protein tyrosine phosphatases

## Abstract

Enzymes are dynamic entities, and their conformational dynamics are intimately linked to their function and evolvability. In this context, protein tyrosine phosphatases (PTPs) are an excellent model system to probe the role of conformational dynamics in enzyme function and evolution. They are a genetically diverse family of enzymes, with a highly conserved catalytic domain, identical catalytic mechanisms, and turnover numbers that vary by orders of magnitude, with their activity being determined by the mobility of a catalytic loop that closes over the active site and places a key catalytic residue in place for efficient catalysis. From a biological perspective, PTPs are important regulators of a host of cellular processes, including cellular signaling, which has made them in particular important anticancer drug targets, among other diseases of interest. The high structural conservation of their active sites renders them therapeutically elusive, but there exist allosteric inhibitors that exploit the allosteric regulation of these enzymes to impede the motion of their catalytic WPD-loops, thus inactivating them. Conformational dynamics and allostery are problems that are ideal for computational investigation, and indeed, advances in computational methodologies have resulted in a range of exciting studies illuminating the molecular details of structure–function–dynamics–allostery links in these enzymes. This review provides both a brief history of computational work in this space, as well as discussing in detail recent advances, illustrating how molecular simulations have been successfully exploited to enhance our fundamental understanding of these biomedically important enzymes, and of the function and regulation of ‘loopy’ enzymes more broadly.

## Introduction

**Figure 1:**
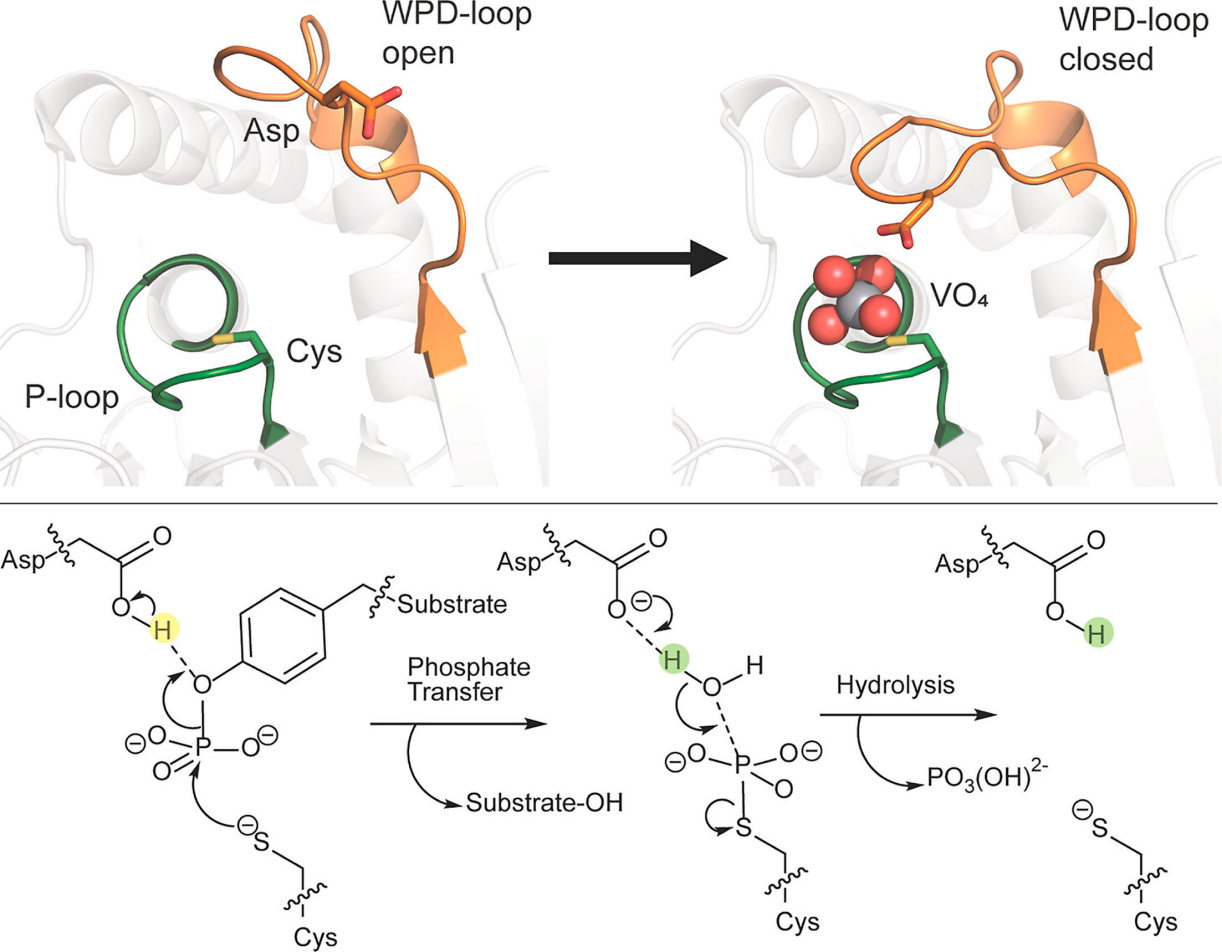
Illustration of (top) WPD-loop closure and (bottom) the conserved catalytic mechanism in protein tyrosine phosphatases. The WPD-loop, carrying the catalytic aspartic acid, is shown in orange, and the phosphate-binding P-loop, carrying the nucleophilic cysteine, is shown in green. The reaction proceeds *via* a two-step cleavage (phosphate-transfer) and then hydrolysis mechanism, with the catalytic aspartic acid on the WPD-loop acting as an acid-base catalyst. This figure is reproduced with permission from ref [14]. . Copyright 2022, Royal Society of Chemistry. Originally published under a CC-BY-NC 3.0 license.

Enzymes are dynamic entities, and the role of the motion of their catalytic loops in enzyme catalysis and evolution, as well as the exploitation of such loop motion for biotechnological purposes, has been a subject of substantive research interest [1–6]. In this context, protein tyrosine phosphatases (PTPs) are an excellent model system with which to probe the role of loop dynamics in catalysis. These enzymes, which hydrolyze phosphorylated tyrosine residues in proteins, have been extensively studied using a barrage of experimental techniques due to their important roles in cellular signaling processes, which makes them important but elusive drug targets (for detailed reviews, see, e.g., refs [7–10]). From a biophysical perspective, PTPs are genetically diverse [11], but with highly conserved catalytic domain structures, identical catalytic mechanisms and transition states, and effectively superimposable catalytic sites [10,12]. Despite these similarities, the turnover numbers for PTPs vary by several orders of magnitude [13], an observation that can be linked to the motion of a key catalytic loop, the WPD-loop (in some organisms an IPD-loop), which closes over the active site and, in doing so, positions a highly conserved aspartic acid side chain into the active site for efficient acid-base catalysis in the two-step mechanism catalyzed by PTPs [12] (Figure 1). Structural, spectroscopic (NMR), and computational analyses have all implicated the importance of the dynamics of this loop in regulating PTP turnover rates [15–18]. Further, WPD-loop motion is, in turn, allosterically regulated, through allosteric mechanisms that appear to be highly evolutionarily conserved across PTPs [18–20].

Probing conformational motions is a problem that is ideal for biomolecular simulation techniques, and given their biomedical importance, WPD-loop motion in PTPs has been a topic of significant interest for biomolecular simulations, dating back over at least 25 years [21,22]. More recent computational techniques have provided significant insight into the motion of the WPD-loop, its role in PTP catalysis, and its allosteric regulation. This review will focus on recent computational advances in this area, both as a means to expand our understanding of PTP function and regulation, and also as a valuable blueprint for studying enzymes regulated by loop motion more broadly.

## Modeling WPD-loop transitions in PTPs

Tarp fluorescence anisotropy and temperature-jump (T-jump) fluorescent experiments in the PTP YopH (a virulence factor from *Yersinia pestis* [23]), which focus on the environment of the conserved Trp on the WPD-loop, W179 (PTP1B)/W354 (YopH), have suggested the presence of WPD-loop kinetics that range from timescales for 3 ns to 3 μs, respectively [24,25]. In contrast, NMR measurements of WPD-loop motion in YopH have shown a timescale of 22 μs for WPD-loop closure in YopH, and an order of magnitude slower transitions in PTP1B [17]. Although molecular dynamics (MD) simulations are ideal for probing questions regarding protein conformational dynamics, such timescales remain, even now, out of reach of conventional molecular dynamics simulations. Therefore, capturing such conformational transitions necessitates more sophisticated enhanced sampling approaches, posing a nontrivial computational challenge [26].

Given computational constraints of the time, early simulation studies of WPD-loop motion in PTPs were extremely short by contemporary standards, on the order of one or a few ns [21,27,28]. Unsurprisingly, on such short timescales, these simulations primarily captured internal dynamics of the loop [21,28], or loop transitions on unphysically fast timescales (~4 ns in unliganded YopH [27]). Early targeted MD (tMD) simulations [29] identified a region of PTP1B, the S-loop (residues 198–209 in PTP1B numbering), that connects the α3-helix adjacent to the WPD-loop and the β-strand connected to the P-loop, that undergoes a concerted conformational change with WPD-loop motion [30] (note that this loop has also been referred to as loop L14 in recent literature [31]). The S-loop takes a knot-like conformation in the WPD-loop open conformation of PTP1B (PDB ID: 2HNP [32]) and unwinds as the WPD-loop transitions to a closed conformation (PDB ID: 1PTV [33]). Both the tMD [29] and subsequent short-timescale conventional MD simulations [28,34] suggested the S-loop is a target for drug discovery, including for allosteric inhibitors [35] that affect the flexibility of this loop (and by doing so block the motion of the WPD-loop). Molecular simulations also highlighted the importance of the highly mobile α7-helix of PTP1B in maintaining WPD-loop stability [36], and the coupling between catalytic loop motions and enzyme global dynamics more broadly [37], among other features [38–42].

**Figure 2:**
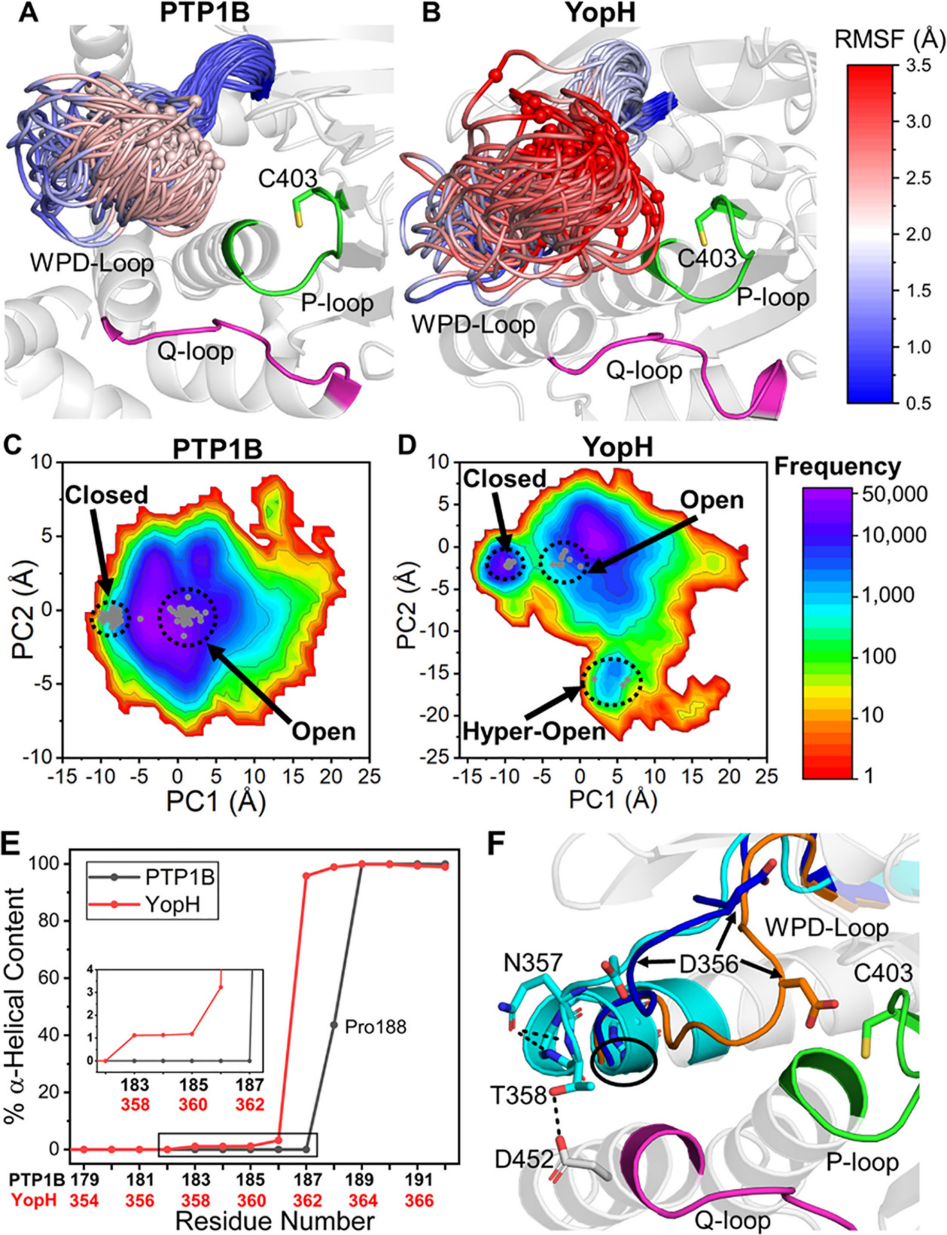
Modeling the conformational dynamics of the catalytic loops of PTP1B and YopH. (**A, B**) Differences in the conformational space sampled by the WPD-loops of (**A**) PTP1B and (**B**) YopH, illustrated by snapshots taken from HREX-MD [43] simulations of each enzyme, colored by the root mean square fluctuations (RMSF) of the WPD-loop C_α_ atoms during the simulations. (**C, D**) 2D histograms constructed as a function of the first two PCs obtained from principal component analysis (PCA) performed on the HREX-MD simulations of (**C**) PTP1B and (**D**) YopH. The corresponding crystal structures of PTP1B and YopH in WPD-loop open and closed conformations are projected onto the respective histograms for reference, and shown as gray dots. (**E**) % helical content of the PTP1B/YopH WPD-loops and adjacent residues during the simulations. (**F**) Illustration of a representative structure of wildtype YopH in a hyper-open WPD-loop from the HREX-MD simulations, showing the extension of the α-helix adjacent to the WPD-loop and the associated stabilizing interactions. This figure was originally published in ref [18], under a CC-BY 4.0 license. Copyright © 2021 the Authors. Published by the American Chemical Society.

More recently, our group combined conventional molecular dynamics simulations, Hamiltonian replica exchange molecular dynamics simulations (HREX-MD) [43], parallel-tempered metadynamics simulations in the well-tempered ensemble (PT-MetaD) [44], and empirical valence bond simulations [45] of chemical reactivity, to study the links between loop dynamics and catalysis in PTPs, focusing on PTP1B and YopH [18]. YopH is ~an order of magnitude more active than PTP1B, with similarly faster motion of the WPD-loop [17]. The calculated barriers to the chemical step for catalysis were found to be similar in these two enzymes [18] and unlikely to account for the observed difference in activity. In contrast, we observed significant differences in the dynamics of the WPD-loop, strongly suggesting that the observed differences in activity are driven by dynamical effects. In agreement with NMR data [17], our HREX-MD [43] simulations indicated that the YopH WPD-loop samples a wider array of conformations, and at a faster rate, than PTP1B (Figure 2). Conventional MD simulations at the reactant state indicated that the large-scale motion of the WPD-loop is supported by faster local side chain fluctuations on the nanosecond timescale, again in agreement with prior NMR data [17]. PT-MetaD simulations allowed us to quantify the free energy differences between the open and closed states of the WPD-loop in liganded and unliganded PTP1B and YopH, showing that the WPD-loop closed conformation was consistently more favorable in YopH than in PTP1B. The PT-MetaD simulations also allowed us to showcase the importance of a second loop, the ‘E-loop’, that moves in concert with the WPD-loop, and regulates its motion, while also providing interactions that stabilize the transition state during catalysis [18]. This is in agreement with both prior NMR and structural studies of PTPs [8,46,47], as well as studies of other ‘loopy’ proteins such as various TIM barrel enzymes with co-ordinated loop motion [26,48–51], indicating the importance of ‘auxiliary’ loops in driving these complex conformational changes over the active site.

Most curiously, prior experimental work [13] had shown that chimeric WPD-loop swapped YopH-PTP1B chimeras (parts of the PTP1B WPD-loop grafted onto the YopH scaffold) adopted a hyper-open conformation of the WPD-loop that has also been observed in other PTPs such as PTPRG, STEP, LYP, and GLEP1 [52,53]. Although catalytically irrelevant, the recurrence of this hyper-open conformation across PTPs suggests it has some functional relevance, which we hypothesized is linked to the allosteric regulation of these enzymes [18]. Although this conformation has never been observed in wildtype YopH, our HREX-MD simulations were able to capture this conformation in wildtype YopH simulations, albeit as a rare event (only ~1% of simulation time). This suggests that this conformation was not ‘created’ by the YopH-PTP1B chimeras but rather was intrinsic to the YopH sequence. Further analysis of our simulations (Figure 2) indicated that the presence of this hyper-open conformation is facilitated by the incorporation of part of the C-terminal portion of the YopH WPD-loop into the adjacent α-helix [18,54], which is not possible in PTP1B due to the WPD-loop P188 acting as a ‘helix breaker’ [18]. This also shows how a single residue can significantly affect the dynamics of the WPD-loop.

Following our curious observations with YopH-PTP1B chimeras, we engaged in joint experimental and computational work to create reverse PTP1B-YopH chimeras, where the WPD-loop of YopH is transposed into PTP1B, as well as a series of intermediate chimeras that systematically restored the native PTP1B loop sequence, one residue at a time [14]. Of these enzymes, four were soluble, and these were subjected to detailed biochemical, structural, and computational characterization. Curiously and interestingly, the resulting chimeric proteins showed significant differences in stability and conformational plasticity both relative to each other and relative to either parent enzyme. Further, in contrast to the YopH-PTP1B chimeras [13], we instead observed a tendency of the WPD-loop to adopt a closed conformation when incorporating the YopH WPD-loop into the PTP1B scaffold [14]. In this sense, the WPD-loops of the chimeric proteins tended to take on exaggerated versions of the native WPD-loop in each parent scaffold, rather than the WPD-loop being incorporated, indicating that regulation of loop motion expands beyond the sequence of the WPD-loop itself and is controlled by the full protein scaffold (see further discussion in the section on **Modeling Loop Dynamics and Allostery in Protein Tyrosine Phosphatases**).

As a more substantive example of how conservative changes in sequence can radically affect protein properties, we recently generated an engineered archaeal PTP, ShufPTP, by manually shuffling the sequences of six thermophilic archaeal parents [55]. While ShufPTP retained high sequence similarity (as high as 90%) to its parent sequences, which in turn all came from organisms with optimal growth temperatures in the range of 80–88 °C [56–60], ShufPTP showed a denaturation temperature >130°C, a rigid acid-loop (IPD- instead of WPD-loop) with significant phosphate binding loop (P-loop) dynamics, multiple oxidation states of the active site cysteine, and a back-up catalytic mechanism [55]. Although these properties had previously been observed to some extent in other characterized extant archaeal PTPs [61,62], they are greatly exaggerated in ShufPTP, showing how even seemingly small jumps in sequence space can have significant biophysical impact [55].

Following from this, in other significant computational studies of WPD-loop dynamics and catalysis, recent long-timescale unbiased molecular dynamics simulations and accelerated weighted ensemble [63] simulations of PTP1B identified a conserved sequence on the WPD-loop, the PDFG motif, that acted as a conformational switch driving transitions between open and closed states of the loop [64]. Further, combined molecular dynamics and string method simulations [65] have demonstrated the importance of friction to WPD-loop dynamics in PTP1B, suggesting that loop motion is governed by torsional rearrangements within the loop, as well as friction caused by backbone adjustments during loop motion, which in turn can dynamically trap the loop and slow its motion [66]. Finally, combined structural and simulation analysis of an atypical plant and fungal PTP from *Arabidopsis thaliana* that lacks an Asp or Glu on the canonical WPD-loop demonstrated that in the absence of this residue, this PTP replaces acid/base catalysis from a loop residue with intramolecular proton transfer facilitated by the substrate [67].

## Modeling loop dynamics and allostery in PTPs

PTPs are allosterically regulated enzymes [19,68]; targeting this allosteric regulation is a promising strategy to surmount the challenges associated with drugging these elusive proteins [69]. Allosteric regulation more broadly is central to biochemistry (famously described by Monod as the ‘second secret of life’ [70]), and unsurprisingly, there has been substantive experimental and computational effort dedicated to understanding protein allostery [71].

There have been numerous elegant experimental studies of allostery in PTPs, many of which have recently been reviewed in ref [72]. As some examples, both multitemperature X-ray crystallography [73] and hydrogen-deuterium exchange mass spectroscopy (HDX-MS) [74] have played an important role in expanding the allosteric network of PTP1B and revealing novel allosteric sites that can be exploited in drug discovery [75–78]. Combined NMR, spectroscopic, biochemical, and simulation analysis of PTP1B allowed for a detailed regulatory network of PTP1B to be constructed, highlighting both the importance of conformational rigidity and dynamic allostery in regulating WPD-loop motion in PTP1B [68]. A subsequent NMR study used single alanine mutations on WPD-loop residues in the PTPs PTP1B and VHR to identify hidden allosteric networks in both enzymes [79]. This was achieved by exploiting the reciprocity [80] that exists in allosteric systems to explore coupling between the orthosteric and allosteric sites, and how this coupling is affected by distal residues. Finally, more recent work has combined solution NMR spectroscopy and microsecond molecular dynamics simulations to show how distal mutations in PTP1B can rewire allosteric networks to control substrate specificity [81].

Further integrated experimental work that combined structural biology, NMR spectroscopy, and pre-steady-state kinetics made a substantive contribution to our understanding of allostery in PTP1B by demonstrating strong correlation between the rate of dephosphorylation of the phosphocysteine enzyme intermediate of PTP1B (Figure 1) and millisecond motions in the WPD-loop and adjacent α-helical domain [82]. This both shows how WPD-loop motion is controlled not just by residues within the loop itself but also by the surrounding protein architecture, providing direct experimental evidence for a link between WPD-loop motions and catalysis. In parallel, both computational and experimental studies have shown that this allosteric regulation is spread across the entire protein scaffold [83], involving, for instance, significant contributions from the C-terminal α7-helix of PTP1B and the closely related TCPTP to regulate loop motions [84,85]. There are also indications that dynamic allostery in PTPs is not yet fully understood: fragment soaking crystallography experiments identified similar allosteric sites in PTP1B and VHR that may affect activity through an unknown mechanism [75,79,86].

**Figure 3:**
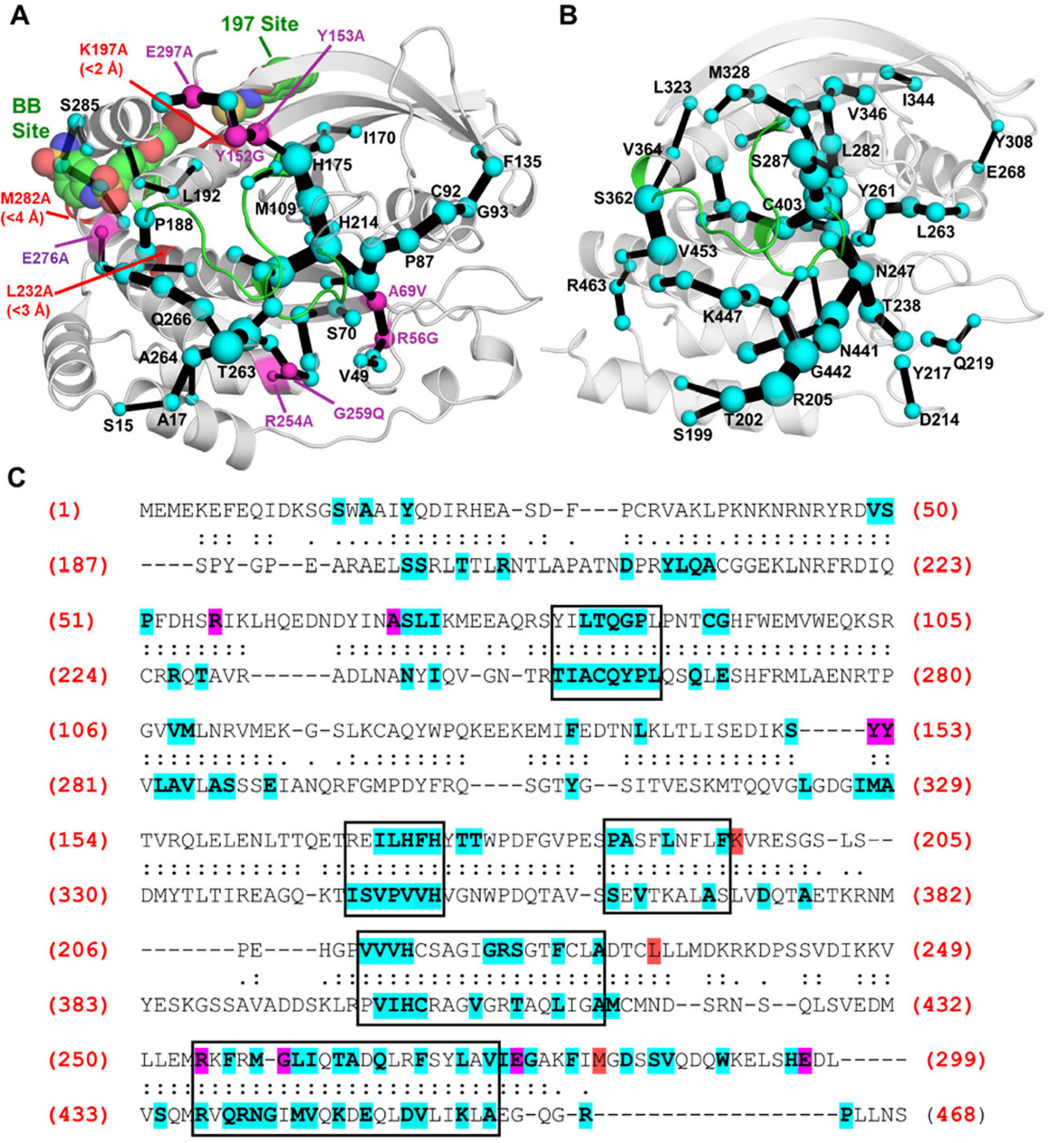
Allosteric communication pathways in PTP1B and YopH. (**A, B**) Allosteric communication pathways in (**A**) PTP1B and (**B**) YopH, as calculated from molecular dynamics simulations using the shortest path map (SPM) approach [96]. The two known allosteric binding sites of PTP1B in panel (**A**) are illustrated by inhibitors bound shown as van der Waals sphere. Further, in the case of PTP1B, residues where mutations are known to alter activity by >|50%| at positions neither on the WPD-loop nor P-loop are shown in purple if on the calculated SPM path, and red if not. As can be seen, the tree structure in PTP1B and YopH is visually similar. (**C**) Structure-based sequence alignment of PTP1B and YopH, with all SPM residues highlighted using the same coloring scheme as in panel (**A**). Boxes indicate regions with a high frequency of SPM residues in both PTP1B and YopH. 35 of the 69 SPM residues identified in PTP1B (~50%) are conserved in YopH, despite the two enzymes having only 20.6% sequence identity. Further details are provided in ref [18]. This figure was originally published in ref [18], under a CC-BY 4.0 license. Copyright © 2021 the Authors. Published by the American Chemical Society.

From an evolutionary perspective, sequence-based statistical analysis has shown the role of evolutionary conserved residue networks mediating protein allosteric communication [87,88] (although divergence of allostery in protein families has also been reported [89–91]), it has been argued that allostery and enzyme catalysis emerge *via* a common route [92], and that allostery plays an important role in protein evolvability [93–95]. In the context of PTPs, structural and computational analysis has suggested strong conservation of allostery among PTPs [18–20,31] (Figure 3) but with unique dynamical and allosteric ‘signals’ in individual enzymes [20,97]. It is clear that restraining WPD-loop motion is a conserved strategy for PTP activity regulation, but the mechanisms that achieve this may differ. For instance, residues of the PTPRA D2 domain directly interact with WPD-loop residues on the D1 domain, limiting its flexibility in a manner distinct from PTP1B’s allosteric network [98]. In SHP2, a unique conformational change of a single buried residue precludes WPD-loop closure [99].

Given the importance of allostery to both enzyme evolution and drug discovery targeting PTPs, being able to characterize and predict allosteric networks in PTPs (and other enzymes) in a reliable fashion significantly improves our understanding of these enzymes, as well as our ability to identify novel potential druggable sites [19,100] and cryptic pockets for drug discovery [101].

## WPD-loop dynamics in PTPs and the pH dependency of catalysis

The pH dependence of catalysis is a standard metric of enzyme activity in a laboratory setting, and in PTPs, this pH dependence is typically bell-shaped, with a pH dependence that ranges from ~4.5 in a hyperthermophilic archaeal PTP [61], to ~7.5 in a mammalian PTP [102], likely reflecting optimization for different biological roles and environments. In recent work, we have combined structural, kinetic, and computational characterization to explore the pH dependence of catalysis in wildtype and mutant forms of the PTPs PTP1B, YopH, and SHP1 [103,104]. Curiously, depending on the enzyme, we observe different but complementary strategies for modulating the pH dependency of catalysis in these enzymes.

**Figure 4:**
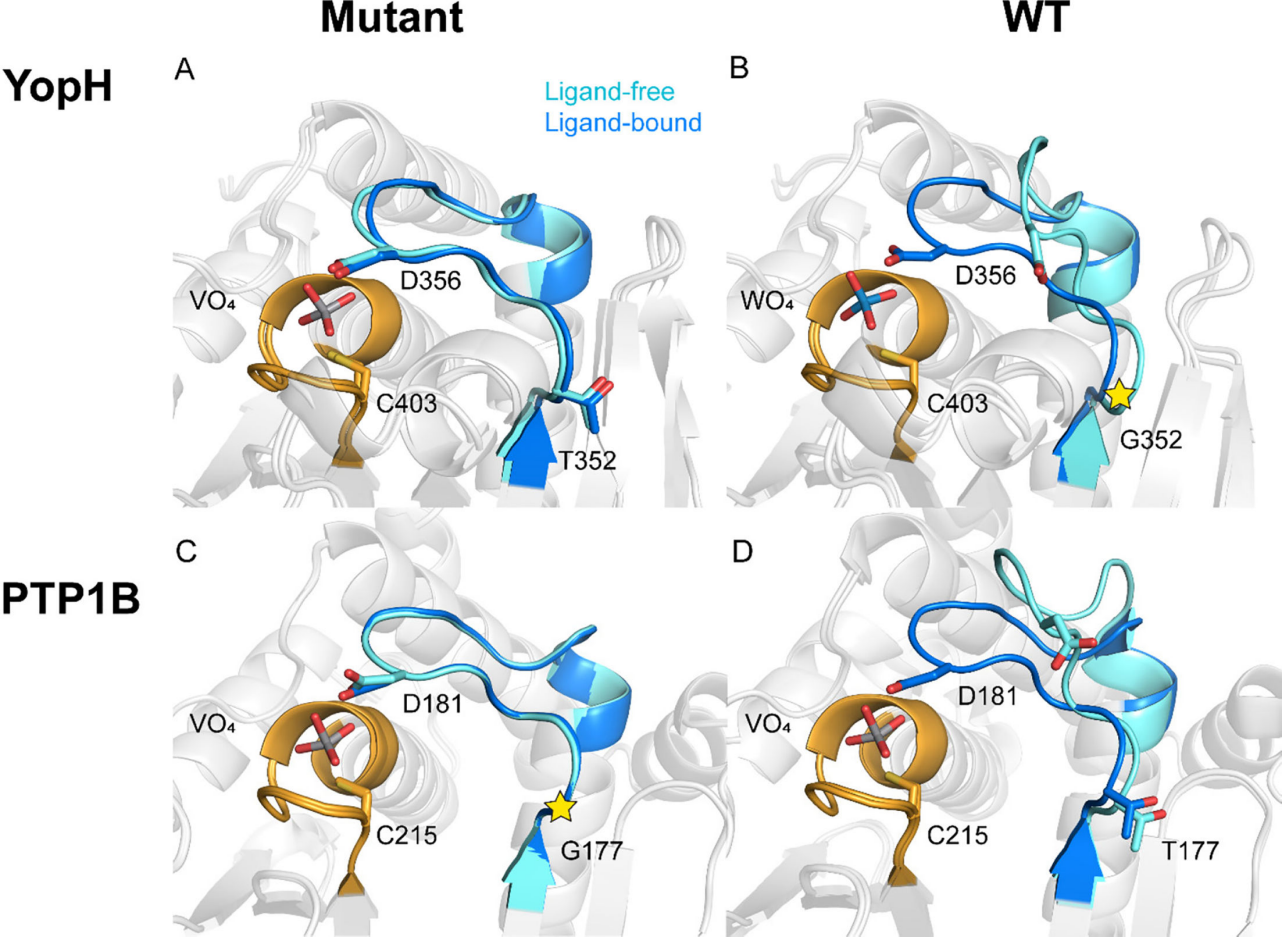
Modulating WPD-loop dynamics through a single point mutation. Comparison of the crystal structures of (**A, C**) mutant and (**B, D**) wildtype (**A, B**) YopH and (**C, D**) PTP1B, comparing the wildtype and T177G and G352T variants of PTP1B and YopH, respectively. Unliganded forms of each enzyme are shown in cyan, and VO_4_-bound forms of each enzyme are shown in blue. The position of the substitution is highlighted by a star in panels (**B**) and (**C**). Introduction of the T177G and G35T substitutions in PTP1B and YopH causes a population shift in the WPD-loop and shifts the equilibrium toward a WPD-loop-closed conformation even in the unliganded form of the enzyme. This, in turn, causes a substantive shift in the pH dependency of catalysis. This figure was originally published in ref [103], under a CC-BY 4.0 license. Copyright © 2021 the Authors. Published by the American Chemical Society.

Specifically, we have explored point mutations of PTP1B, YopH, and SHP1 using combinations of structural, biochemical, and computational characterization [103,104]. We observe that in PTP1B and YopH, a single point mutation (WPD-loop substitutions T177G in PTP1B, G325T in YopH) significantly affects the pH dependency of catalysis (Figure 4), reflected primarily as a shift in the kinetic p*K*_a_ of the active site cysteine, which simultaneously undergoes only a modest shift in its thermodynamic p*K*_a_, based on titration experiments [103]. Molecular simulations indicate that this shift is the result of altered conformational dynamics of the WPD-loop, which in the mutants favors a closed conformation in both liganded and unliganded forms of the enzyme [103]. Thus, in these enzymes, conformational dynamics is affecting the pH dependence of catalysis, without changes in interactions to either the active site cysteine nucleophile on the P-loop, or the catalytic aspartic acid on the WPD-loop [103]. This provides a means for nature to regulate the pH-dependency of catalysis without affecting thermodynamic p*K*_a_s, which depend on the electrostatic environments of the surrounding residues [105]. In contrast, in the PTP SHP1, the impact of point mutations on the WPD-loop (H422Q, E427A, and S418A) on the pH-dependency of catalysis is far more modest and is suggested by simulations to result from subtle changes in solvation networks and H-bonding interactions with the acid/base catalyst on the WPD-loop, D421, rather than substantive dynamical effects on the loop [104]. These contrasting results showcase distinct strategies used by these enzymes to control the pH dependency of catalysis, which in turn is a reflection of how PTPs from different organisms can rapidly biochemically adapt to environmental changes.

## Conclusions

PTPs form an important family of signaling enzymes that have been heavily studied both out of biochemical interest and for their role as drug targets for human disease, particularly cancer [7–10]. There has been substantive experimental and computational effort outlined in elucidating the mechanisms of catalysis and regulation in these enzymes, as well as identifying novel allosteric sites to target in drug discovery efforts [7–10]. This review focuses on providing a brief history of computational efforts in this space, focusing on exciting and ongoing computational developments, both from our own lab and that of others. In particular, as outlined here, substantive advances in both method development to model complex conformational changes, as well as to characterize protein allosteric networks, has significantly opened doors to computationally understand these challenging-to-model enzymes. Although beyond the scope of the current review, computational developments have also allowed water networks in PTPs to be extensively characterized [106], and exascale simulations have shown significant promise in allowing for classification of the pathogenicity of PTP missense VUS (variants of uncertain significance) [107]. These advances, in turn, have expanded our understanding of function, loop dynamics, allostery, and evolution in these enzymes, insights that are relevant not just to PTPs, but to understanding loopy enzymes more broadly.

## Open Access

Open access for this article was enabled by the participation of Lund University in an all-inclusive Read & Publish agreement with Portland Press and the Biochemical Society under a transformative agreement with BIBSAM.

## Abbreviations

HDX-MS: hydrogen-deuterium exchange mass spectroscopy
HREX-MD: Hamiltonian replica exchange - molecular dynamics simulations
MD: molecular dynamics
PTPs: protein tyrosine phosphatases
VUS: variants of uncertain significance
